# Supplementary material for: Social contact patterns during the COVID-19 pandemic in 21 European countries – evidence from a two-year study
Source: BMC Infect Dis. 2023 Apr 26;23:268. doi: 10.1186/s12879-023-08214-y (PMC10132446; doi:10.1186/s12879-023-08214-y)
Supplement: Supplementary file 1 — Additional file 1: Supplementary Material I. Supplementary Material II. Characteristics of survey responses in 17 G123 countries. Supplementary Material III. Crude daily mean daily number of contacts without censoring by setting and country. [file 12879_2023_8214_MOESM1_ESM.docx]

# Supplementary Material I


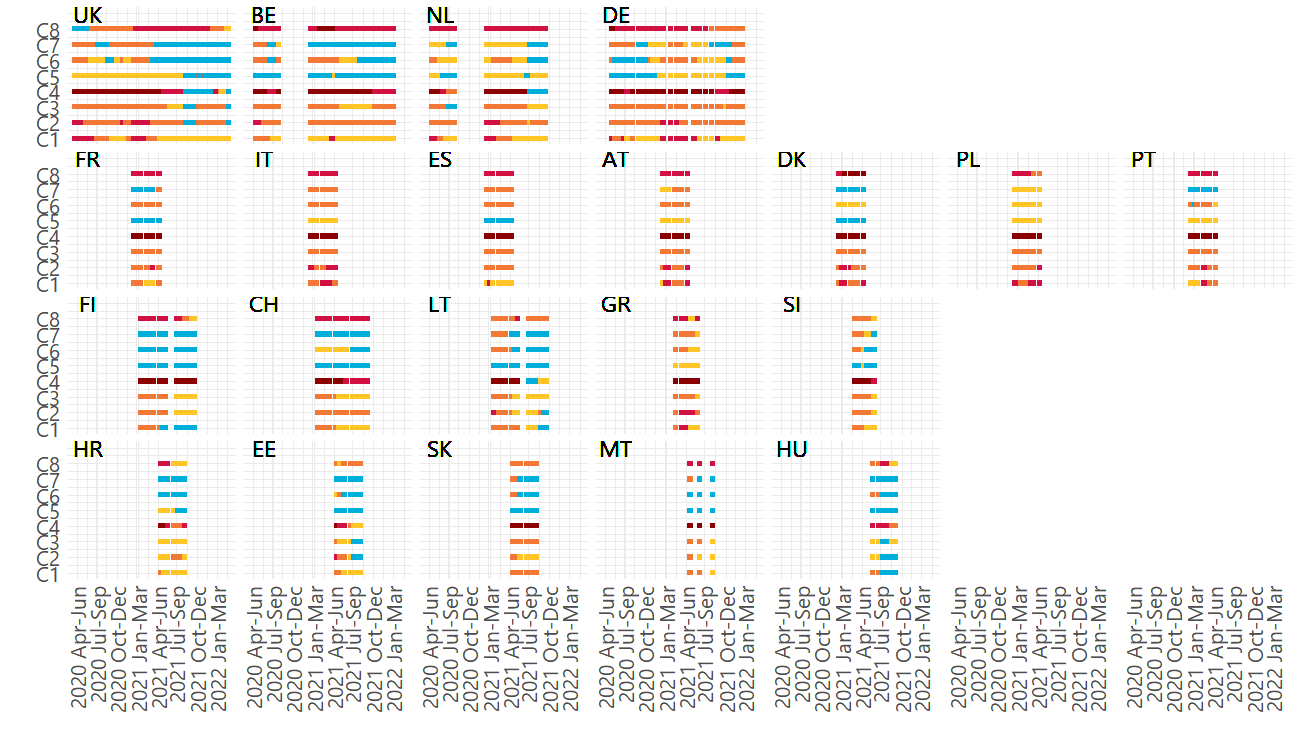


| **Containment and closure policies** | | **Coding** |
| --- | --- | --- |
| C1 | Closings of schools and universities | ∎ No measures |
|  |  | ∎ Recommend closing or all schools open with alterations |
|  |  | ∎ Require closing |
|  |  | ∎ Require closing all levels |
| C2 | Closings of workplaces | ∎ No measures |
|  |  | ∎ Recommend closing or all businesses open with alterations |
|  |  | ∎ Require closing for some sectors or categories of workers |
|  |  | ∎ Require closing for all-but-essential workplaces |
| C3 | Cancelling public events | ∎ No measures |
|  |  | ∎ Recommend cancelling |
|  |  | ∎ Require cancelling |
| C4 | limits on gatherings | ∎ No restrictions |
|  |  | ∎ Restrictions on very large gatherings (the limit is above 1000 people) |
|  |  | ∎ Restrictions on gatherings between 101-1000 people |
|  |  | ∎ Restrictions on gatherings between 11-100 people |
|  |  | ∎ Restrictions on gatherings of 10 people or less |
| C5 | losing of public transport | ∎ No measures |
|  |  | ∎ Recommend closing |
|  |  | ∎ Require closing |
| C6 | Orders to confine to the home | ∎ No measures |
|  |  | ∎ Recommend not leaving house |
|  |  | ∎ Require not leaving house with exceptions for daily exercise, grocery shopping, and 'essential' trips |
|  |  | ∎ Require not leaving house with minimal exceptions |
| C7 | Restrictions on internal movement | ∎ No measures |
|  |  | ∎ Recommend not to travel between regions/cities |
|  |  | ∎ Internal movement restrictions in place |
| C8 | Restrictions on international travel | ∎ No restrictions |
|  |  | ∎ Screening arrivals |
|  |  | ∎ Quarantine arrivals from some or all regions |
|  |  | ∎ Ban arrivals from some regions |
|  |  | ∎ Ban on all regions or total border closure |

# Supplementary Material II

Characteristics of survey responses in 17 G123 countries

|  | at | ch | dk | ee | es | fi | fr | gr | hr | hu | it | lt | mt | pl | pt | si | sk |
| --- | --- | --- | --- | --- | --- | --- | --- | --- | --- | --- | --- | --- | --- | --- | --- | --- | --- |
| respondents | 1737 | 4425 | 1728 | 1852 | 1622 | 2622 | 1704 | 2240 | 1971 | 2078 | 1616 | 2593 | 1702 | 1724 | 1839 | 1998 | 1733 |
| responses | 7025 | 12149 | 7044 | 6967 | 7048 | 11784 | 7025 | 7111 | 7080 | 7207 | 7048 | 12763 | 2759 | 7011 | 7018 | 7510 | 7048 |
| 18-29 | 18.5 | 15.6 | 14.1 | 13.1 | 14.6 | 14.8 | 14.9 | 16.1 | 18.4 | 12.4 | 15.0 | 14.9 | 17.3 | 18.8 | 16.0 | 15.8 | 15.3 |
| 30-39 | 17.2 | 18.5 | 10.5 | 16.9 | 16.9 | 15.3 | 17.4 | 21.1 | 16.4 | 16.9 | 13.5 | 16.5 | 24.8 | 19.3 | 16.1 | 20.3 | 20.5 |
| 40-49 | 16.7 | 16.3 | 13.7 | 16.6 | 22.5 | 15.5 | 16.5 | 26.3 | 20.1 | 22.2 | 21.8 | 16.9 | 20.8 | 19.2 | 21.8 | 21.4 | 20.6 |
| 50-59 | 19.6 | 20.2 | 22.5 | 18.4 | 17.7 | 17.1 | 15.3 | 24.4 | 18.1 | 16.5 | 15.7 | 18.7 | 16.2 | 18.0 | 19.2 | 20.9 | 16.5 |
| 60-69 | 18.5 | 17.2 | 22.8 | 18.7 | 18.8 | 23.0 | 22.4 | 10.1 | 21.6 | 22.4 | 20.5 | 23.4 | 14.5 | 18.9 | 18.4 | 18.1 | 19.9 |
| 70-120 | 9.5 | 12.1 | 16.4 | 16.3 | 9.4 | 14.3 | 13.4 | 2.0 | 5.5 | 9.7 | 13.5 | 9.6 | 6.5 | 5.8 | 8.5 | 3.5 | 7.2 |
| female | 47.0 | 49.0 | 47.2 | 54.2 | 49.6 | 47.2 | 46.6 | 48.7 | 51.4 | 47.4 | 46.6 | 52.9 | 57.8 | 55.6 | 45.6 | 56.8 | 47.4 |
| male | 53.0 | 51.0 | 52.8 | 45.8 | 50.4 | 52.8 | 53.4 | 51.3 | 48.6 | 52.6 | 53.4 | 47.1 | 42.2 | 44.4 | 54.4 | 43.2 | 52.6 |
| old | 14.7 | 14.5 | 17.4 | 19.8 | 25.7 | 16.8 | 19.8 | 15.1 | 23.6 | 20.7 | 33.2 | 18.9 | 17.4 | 20.8 | 21.2 | 17.0 | 20.8 |
| hh_size | 2.3 | 2.3 | 2.1 | 2.3 | 2.8 | 2.0 | 2.4 | 2.8 | 2.9 | 2.6 | 2.8 | 2.5 | 3.0 | 3.0 | 2.6 | 2.8 | 2.7 |
| likely | 3.1 | 4.9 | 2.8 | 9.8 | 6.0 | 2.1 | 10.4 | 15.7 | 7.2 | 4.1 | 3.6 | 23.5 | 6.0 | 6.9 | 9.5 | 4.5 | 2.8 |
| serious | 10.5 | 9.3 | 12.8 | 20.0 | 26.1 | 16.8 | 14.3 | 18.1 | 14.6 | 13.3 | 15.6 | 17.7 | 19.6 | 22.5 | 23.9 | 13.9 | 9.4 |
| spread | 11.2 | 14.8 | 29.6 | 13.7 | 55.4 | 13.3 | 25.0 | 30.4 | 30.2 | 11.1 | 24.2 | 25.9 | 30.2 | 23.2 | 50.3 | 23.4 | 16.7 |
| mask | 68.2 | 76.7 | 74.4 | 49.9 | 90.5 | 65.3 | 84.9 | 89.8 | 85.3 | 36.8 | 89.0 | 75.3 | 94.6 | 85.5 | 83.8 | 72.5 | 78.4 |
| vacc | 3.8 | 20.3 | 3.5 | 30.7 | 2.8 | 25.3 | 5.6 | 11.2 | 22.4 | 35.1 | 3.7 | 24.1 | 60.5 | 4.2 | 2.5 | 10.7 | 23.3 |
| risk | 22.2 | 22.7 | 22.9 | 32.5 | 31.8 | 31.7 | 25.9 | 19.2 | 28.5 | 33.1 | 21.7 | 33.8 | 19.7 | 35.6 | 26.0 | 24.7 | 31.0 |
| symp_fever | 1.0 | 1.6 | 1.2 | 1.4 | 1.6 | 2.2 | 2.3 | 1.5 | 1.9 | 1.6 | 1.7 | 1.5 | 0.8 | 2.6 | 0.8 | 1.6 | 1.5 |
| symp_cough | 5.4 | 4.9 | 7.0 | 3.7 | 7.8 | 5.2 | 5.1 | 4.6 | 4.9 | 6.6 | 4.9 | 3.6 | 1.6 | 10.1 | 5.2 | 6.1 | 4.5 |
| symp_sob | 2.3 | 2.6 | 3.0 | 1.8 | 2.5 | 3.0 | 2.7 | 1.6 | 2.2 | 2.3 | 2.2 | 1.6 | 1.2 | 2.9 | 1.6 | 2.3 | 3.4 |
| symp_ache | 14.8 | 13.8 | 16.9 | 13.9 | 21.9 | 16.8 | 15.8 | 13.7 | 12.9 | 16.7 | 15.6 | 10.2 | 12.7 | 23.0 | 14.6 | 12.2 | 14.7 |
| symp_congestion | 10.5 | 6.5 | 9.5 | 8.3 | 9.2 | 14.2 | 7.5 | 6.1 | 7.3 | 7.5 | 6.0 | 6.3 | 4.0 | 13.5 | 7.4 | 8.6 | 5.2 |
| symp_sore_throat | 3.8 | 3.5 | 4.2 | 2.9 | 5.4 | 3.7 | 4.5 | 3.1 | 2.9 | 3.8 | 4.8 | 3.2 | 1.6 | 6.9 | 3.4 | 3.8 | 4.1 |
| symp_tired | 6.4 | 5.3 | 4.5 | 4.2 | 4.5 | 5.3 | 4.4 | 5.3 | 3.7 | 6.7 | 4.2 | 4.3 | 3.6 | 8.5 | 3.3 | 4.0 | 5.7 |
| symp_any | 28.6 | 24.8 | 28.4 | 24.6 | 34.4 | 30.1 | 27.1 | 23.6 | 22.6 | 27.2 | 26.6 | 18.3 | 18.9 | 37.6 | 23.5 | 22.4 | 23.8 |

# Supplementary Material III

Crude daily mean daily number of contacts without censoring by setting and country

|  | **UK** | **BE** | **NL** | **DE** |  |  |  |
| --- | --- | --- | --- | --- | --- | --- | --- |
| **All** | 4.01 (3.86,4.16) | 5.36 (4.75,5.97) | 5.39 (3.75,7.03) | 2.95 (2.77,3.12) |  |  |  |
| **Home** | 1.16 (1.01,1.30) | 1.51 (0.90,2.13) | 1.20 (-0.44,2.84) | 0.96 (0.79,1.14) |  |  |  |
| **Work** | 1.31 (1.16,1.46) | 2.01 (1.40,2.63) | 2.38 (0.74,4.02) | 0.62 (0.45,0.80) |  |  |  |
| **Others** | 1.55 (1.40,1.70) | 1.84 (1.22,2.45) | 1.81 (0.17,3.45) | 0.81 (0.63,0.98) |  |  |  |
|  | **ES** | **FR** | **IT** | **AT** | **DK** | **PO** | **PL** |
| **All** | 3.20 (3.01,3.40) | 3.41 (3.03,3.80) | 3.13 (2.94,3.32) | 2.99 (2.74,3.24) | 3.93 (3.60,4.27) | 4.98 (3.87,6.09) | 5.19 (3.83,6.54) |
| **Home** | 1.69 (1.50,1.88) | 1.23 (0.85,1.62) | 1.60 (1.41,1.80) | 1.33 (1.08,1.58) | 1.30 (0.97,1.64) | 1.68 (0.57,2.79) | 1.76 (0.41,3.12) |
| **Work** | 0.90 (0.71,1.10) | 1.09 (0.71,1.47) | 0.94 (0.75,1.13) | 0.86 (0.61,1.11) | 1.22 (0.89,1.56) | 1.43 (0.32,2.54) | 1.69 (0.34,3.05) |
| **Others** | 0.62 (0.42,0.81) | 1.10 (0.71,1.48) | 0.59 (0.40,0.78) | 0.81 (0.56,1.06) | 1.41 (1.07,1.75) | 1.87 (0.76,2.98) | 1.74 (0.39,3.10) |
|  | **CH** | **FI** | **LT** | **GR** | **SI** |  |  |
| **All** | 5.52 (4.78,6.27) | 5.32 (4.47,6.17) | 6.28 (4.95,7.60) | 5.29 (2.36,8.23) | 6.23 (4.81,7.64) |  |  |
| **Home** | 1.31 (0.56,2.05) | 1.01 (0.16,1.87) | 1.30 (-0.02,2.63) | 1.63 (-1.30,4.57) | 1.87 (0.45,3.29) |  |  |
| **Work** | 2.34 (1.60,3.09) | 2.25 (1.40,3.10) | 2.21 (0.89,3.54) | 2.58 (-0.36,5.51) | 2.62 (1.20,4.04) |  |  |
| **Others** | 1.88 (1.13,2.63) | 2.06 (1.21,2.91) | 2.77 (1.45,4.09) | 1.10 (-1.84,4.03) | 1.75 (0.33,3.16) |  |  |
|  | **EE** | **HU** | **SK** | **MT** | **HR** |  |  |
| **All** | 6.19 (5.53,6.84) | 4.48 (3.61,5.36) | 5.66 (4.96,6.35) | 8.31 (7.21,9.41) | 6.32 (5.86,6.77) |  |  |
| **Home** | 1.35 (0.69,2.00) | 1.58 (0.71,2.46) | 1.62 (0.93,2.32) | 2.19 (1.09,3.29) | 1.93 (1.47,2.38) |  |  |
| **Work** | 2.46 (1.81,3.12) | 1.75 (0.87,2.62) | 2.09 (1.40,2.79) | 2.59 (1.49,3.69) | 2.28 (1.82,2.74) |  |  |
| **Others** | 2.39 (1.73,3.04) | 1.17 (0.29,2.04) | 1.96 (1.27,2.65) | 3.54 (2.44,4.64) | 2.12 (1.67,2.58) |  |  |

# Supplementary Material IV

[supp_table_forest](https://github.com/wongkerry/epipose_paper_1/blob/main/outputs/forest_plot_est.csv)

# Supplementary Material V

[supp_contacts_factors_by_country](https://github.com/wongkerry/epipose_paper_1/blob/main/outputs/supp_contacts_factors_by_country.pdf)

# Supplementary Material VI

[supp_contact_children](https://github.com/wongkerry/epipose_paper_1/blob/main/outputs/supp_contacts_children.xlsx)
